# Comparison of Linear vs. Cyclic RGD Pentapeptide Interactions with Integrin α_v_β_3_ by Molecular Dynamics Simulations

**DOI:** 10.3390/biology10070688

**Published:** 2021-07-20

**Authors:** Na Li, Simei Qiu, Ying Fang, Jianhua Wu, Quhuan Li

**Affiliations:** 1 School of Bioscience and Bioengineering, South China University of Technology, Guangzhou 510006, China; desertroselina@126.com (N.L.); 201820136121@mail.scut.edu.cn (S.Q.); yfang@scut.edu.cn (Y.F.); wujianhua@scut.edu.cn (J.W.); 2 Guangdong Provincial Engineering and Technology Research Center of Biopharmaceuticals, South China University of Technology, Guangzhou 510006, China; 3 Key Laboratory of Southern Subtropical Plant Diversity, Fairy Lake Botanical Garden, Shenzhen & Chinese Academy of Sciences, Shenzhen 518004, China

**Keywords:** integrin α_v_β_3_, RGD peptide, force-induced dissociation, molecular dynamics simulations

## Abstract

**Simple Summary:**

The integrin α_v_β_3_-RGD motif interaction plays a key role in the progression of malignant tumor. Although two typical cyclic and linear RGD short peptides have been widely used in tumor diagnosis and therapy, little is known about the internal dynamic mechanism for different configurations of RGD peptides with different affinities interacting with the integrin α_v_β_3_. Our results showed that the cyclic RGD peptide had a more stable configuration in binding to integrins α_v_β_3_, which depended on the higher binding energy and higher static electrical energy, especially in the interaction between Asp^RGD^-MIDAS. The steered molecular dynamics simulation showed a stronger interaction for the cyclic RGD-integrin α_v_β_3_ system than the linear one, with a larger dissociation force (average peak force) and more time to dissociate. Our findings provide insights into the dynamics of integrin α_v_β_3_ interactions with linear and cyclic RGD ligands and offer some new therapeutic approaches for the design and development of novel antitumor drugs.

**Abstract:**

Integrin α_v_β_3_ interacting with the short Arg-Gly-Asp (RGD) motif plays a critical role in the progression of several types of tumors. However, the effects of the RGD structure (cyclic or linear) with integrin α_v_β_3_ at the atomic level remain poorly understood. Here, we performed association and dissociation dynamic simulations for integrin α_v_β_3_ in complex with a linear or cyclic pentapeptide by steered molecular dynamics simulations. Compared with cyclic RGD, the linear RGD peptide triggers instability of the configurational changes, mainly resting with the RGD domain due to its flexibility. The main interaction energy between Mg^2+^ and cyclic RGD is much stronger than that of the linear RGD system by the well shield to lessen attacks by free water molecules. The force-dependent dissociation results show that it is easier for linear RGD peptides to leave the active site and much quicker than the cyclic RGD ligand, whereas it is harder to enter the appropriate active binding site in linear RGD. The Ser^123^-Asp^RGD^ bond may play a critical role in the allosteric pathway. Our findings provide insights into the dynamics of α_v_β_3_ interactions with linear and cyclic RGD ligands and contribute to the application of RGD-based strategies in preclinical therapy.

## 1. Introduction

Tumor angiogenesis, the formation of new blood vessels, is a critical process for tumor growth and metastasis [1,2,3]. Many types of adhesion molecules are involved in tumor angiogenesis [4]. Among cell adhesion molecules (CAMs), integrins are important CAMs that link the extracellular matrix (ECM) and the cytoskeleton and participate in adhesive events during various cancer stages, such as tumor growth, invasion, and metastasis [5]. Among all integrins, integrin α_v_β_3_ plays a crucial role in angiogenesis and tumor metastasis, which is widely expressed in many kinds of human tumor biopsy samples but not in vessels in normal tissues [6,7]. As a receptor, integrin α_v_β_3_ can specifically recognize one or more Arg-Gly-Asp tripeptide motifs, called RGD peptides. In the past decade, RGD peptides have been designed to target integrin for cancer therapy [8], and many radiolabeled RGD peptides have been used as α_v_β_3_ -targeting tumor imaging agents [7,9]. In addition, RGD-based anticancer strategies have good prospects in the field of cancer diagnosis and therapy, even in the field of tissue regeneration, including cornea repair, artificial vascularization, and bone tissue regeneration [10,11]. Thus, the use of RGD-modified peptides to inhibit the activity of integrin α_v_β_3_ is a promising strategy for tumor targeting.

Interaction studies of integrin α_v_β_3_ with RGD peptides have greatly advanced our understanding of the dynamic binding process and molecular mechanism [12,13,14]. The activation mechanism of integrin α_v_β_3_ binding to fibronectin has also been revealed to some extent recently [15]. Under different physiological conditions, integrin α_v_β_3_ can form different dynamic conformations with distinct affinity [16]. When the RGD peptide binds to the active region of integrin α_v_β_3_, a shallow crevice is located between the α_v_ and β_3_ subunits, which changes the conformation of integrin and transduces signals from the ECM to the cytoplasm. There are two important interaction sites. One is the salt bridges formed between Arg^RGD^ residue of RGD with Asp^218^ and Asp^150^ residues of the β-propeller subunit, and the other is the Asp-MIDAS interaction, in which Asp^RGD^ carboxylate oxygen atoms of RGD coordinate with the metal ion at the metal ion-dependent adhesion site (MIDAS) of the βA domain. The breaking of the Asp-MIDAS ligand interaction corresponds to the major force peak as the largest barrier to unbinding [17]. The discovery of the structural mechanism of interaction between integrins and RGD-containing peptide ligands has contributed to the rational design of drugs that effectively inhibit integrin activation.

In preclinical studies, various RGD peptides or peptidomimetics are designed as selective integrin inhibitors [9,18,19]. The steric conformation of the peptide and the structural features of the RGD ligand have become important because these factors influence the affinity between RGD and integrin α_v_β_3_. Recent studies have shown that cyclic RGD peptides commonly help improve the binding properties of RGD peptides, whereas linear RGD peptides are easily susceptible to chemical degradation [9,20,21]. Therefore, the cyclic RGD is more stable and more active. However, differences in the dynamic properties of integrin α_v_β_3_ interacting with linear or cyclic RGD peptides remain unknown at the molecular, and even at the atomic, level.

Integrins have been studied as therapeutic targets in many diseases due to their involvement in modulating various vital physiological and pathological processes, including proliferation, survival, differentiation, and migration. Recently, several crystal structures of integrins with or without ligands have been analyzed [22]. Although the details of the molecular interaction mechanism of these structures are not clear, molecular dynamics (MD) simulation may have the ability to derive these insights at the molecular level, which will help better design drugs targeting integrin [23,24]. In this study, we used computational MD and SMD to investigate the binding divergence of different RGD structures (cyclic or linear) interacting with integrin α_v_β_3_. The corresponding structural stability and energy fluctuation profiles were obtained. These results provide insights into the differences in the dynamic properties of RGD-containing peptides with the integrin objective, which will help better understand the field of RGD-mediated drug delivery and imaging constructs.

## 2. Materials and Methods

### 2.1. System Setup

**Figure 1:**
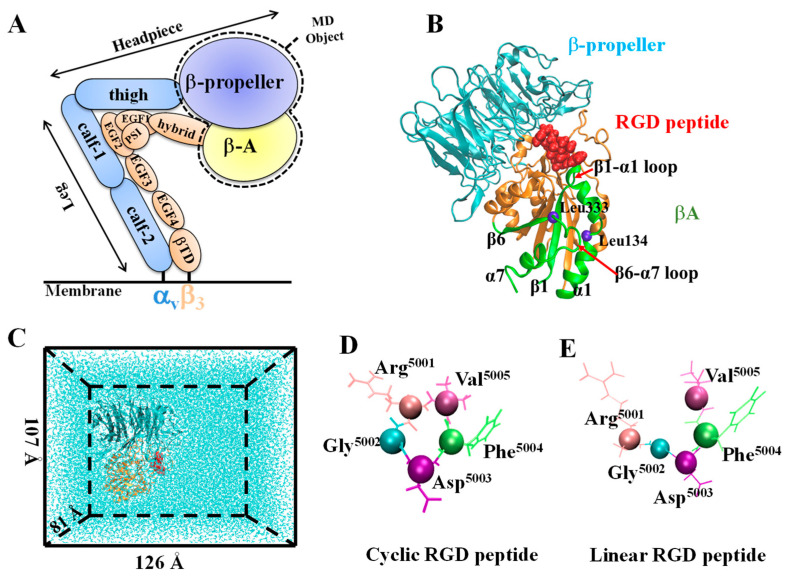
System setup. (**A**) A schematic of the extracellular domain of integrin α_v_β_3_. (**B**) Secondary structure of the integrin headpiece fragments in a NewCartoon representation. β–propeller and βA domains are indicated in cyan and orange, respectively. RGD peptide ligand is shown in red VDWs; the T-junction structure is represented in a green NewCartoon, with β6, α7, β1, and α1 from left to right; the blue sphere is an alpha carbon atom of residues. (**C**) The RGD ligated with integrin α_v_β_3_ headpiece in a water box used for equilibration. (**D**) The cyclic RGD peptide is shown in ResID. (**E**) The linear RGD peptide is shown in ResID. The cyclo-RGDfV and linear RGDfV peptides have the same meaning as the cyclic and linear RGD peptides in this study, respectively.

Two simulated systems of integrin α_v_β_3_ binding with cyclic or linear RGD were set up for the MD simulations (Figure 1). The simulation for the cyclic system was started from the crystal structure 1L5G (resolution: 3.20 Å) containing the complex of integrin α_v_β_3_ headpiece with the cyclic RGD peptide [25]. The linear MD system was then obtained from the same crystal structure but only the RGD sequence was linearized (Figure 1D,E). To reduce the system size, we only used the headpiece of integrin α_v_β_3_, including the βA domain of the β_3_ subunit (residues 110 to 353) and the β-propeller domain of the α_v_ subunit (residues 1 to 438) (Figure 1A,B). Before MD simulations, the complex was solvated in a 126 × 107 × 81 Å^3^ water box together with Na^+^ and Cl^−^ ions to neutralize the system at a 150 mM ionic concentration, resulting in 104,246 atoms (Figure 1C). Due to the lack of Mn^2^^+^ force field parameters, a universal method in which Mn^2^^+^ is replaced by Mg^2+^, metal ion-binding sites were occupied by Mg^2+^ ions in the integrin α_v_β_3_ headpiece in our simulations.

### 2.2. Simulation Procedure and Parameters

All MD simulations were performed with the program NAMD 2.6, using periodic boundary conditions and the CHARMM22 force field [26,27]. The particle-mesh Ewald method was used to calculate the full electrostatic calculations and van der Waals interactions were evaluated using a smooth cutoff (12 Å). Visualization, molecular graphics, and analyses of simulations, including root mean square deviation (RMSD), the distance between two atoms or the centers of mass of the helix, and solvent accessible surface area (SASA) with a 1.4 Å probe radius, were measured in Tcl within the program VMD [28]. A hydrogen bond was defined when the donor–acceptor distance was less than 0.35 nm, and the donor–hydrogen–acceptor angle was less than 30°. Occupancy was evaluated by the proportion of the bond survival time in the simulation time. In all figures, the red and blue curves indicate the linear and cyclic RGD-containing liganded systems, respectively.

Each system was first subjected to energy minimization for two consecutive 50,000 conjugate gradient steps: first with backbone atoms fixed, and second with all atoms free. The energy-minimized structures were then gradually heated from 0 to 310 K and subsequently equilibrated for 10 ns under constant pressure and temperature conditions. The temperature was maintained at 310 K and controlled with Langevin dynamics, and the pressure was maintained at 1 atm using the Langevin piston method.

Constant velocity-steered molecular dynamics simulations (SMD) were performed to accelerate integrin unbinding and started from snapshots sampled after 10 ns of equilibration. We used a time step of 2 fs, a uniform dielectric constant of 1.0, a 12 Å cut-off for non-bonded interactions, and a scaling factor of 1–4 interactions of 1.0. During the SMD simulations, the RGD ligand was pulled away from the binding shallow crevice located between the α_v_ and β_3_ subunits of the integrin α_v_β_3_ headpiece. The C or N termini of the βA and β-propeller subunits were fixed as original points. The pulling potential moved with a constant velocity *v,* and the Cα atom of RGD^5005^ was steered as the SMD atom. In constant-velocity simulations, the time dependence of the external force is *F* = *k*(*vt* − Δ*x*), where Δ*x* is the displacement along the pulling direction at time *t* = 0, *v* is the pulling velocity, and *k* is the spring constant. The rupture force presents the maximum of the force spectrum. Here, we set the pulling speed as *v* = 0.02 Å⋅ps^−1^, 0.04 Å⋅ps^−1^, and 0.06 Å⋅ps^−1^ and the spring constant as *k* = 7 kcal⋅mol^−1^⋅Å^−2^. The SMD simulation was performed in an NVE ensemble at 310 K and lasted for at least 400,000 steps.

In the binding process, the ligand was placed at a distance of 25 Å from the target binding gorge first, and then solvated with a TIP3P water box, which was neutralized at a 150 mM ionic concentration, resulting in a system of 87,098 atoms in a 102 × 94 × 92 Å^3^ water box. After the system minimized and equilibrated, we chose a pushing speed of 0.1 Å⋅ps^−1^ and a spring constant of 2 kcal⋅mol^−1^⋅Å^−2^, and next pushed the RGD ligand into integrin α_v_β_3_ from the direction of the Asp sidechain oxygen OD2 of the ligand to the MIDAS site.

## 3. Results

### 3.1. Configurational Changes for RGD-Integrin α_v_β_3_ Interaction during Equilibration

**Figure 2:**
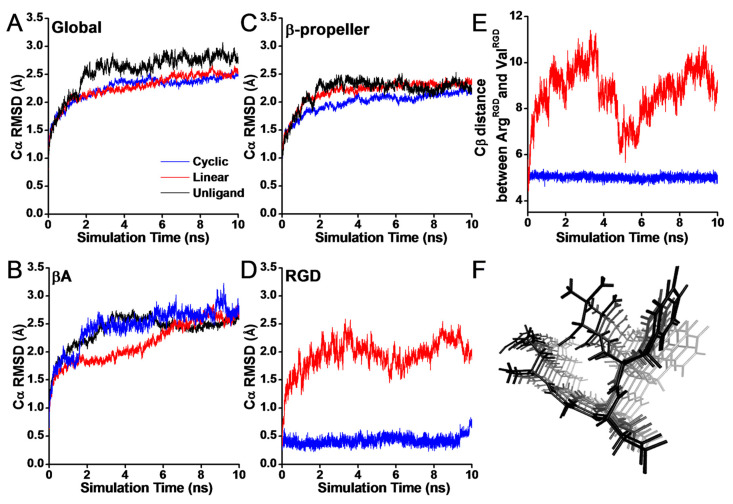
The simulation time curves of RMSD for RGD liganded integrin α_v_β_3_ during equilibration for the global systems (**A**), the β-propeller regions (residues 1–438) (**B**), the βA regions (residues 110–353) (**C**), and the RGD peptide segments (**D**). Each time–RMSD profiles was averaged from three independent runs. The unliganded α_v_β_3_ integrin regions are shown in black, the cyclic RGD and its complex with integrin α_v_β_3_ are shown in blue, and the linear RGD and its complex with integrin α_v_β_3_ are shown in red. (**E**) Time series of the C_β_-atom distance between Arg^RGD^ and Val^RGD^. (**F**) The trajectory of the linear RGD ligand as time changed from dark to light grey. Image of all 50 frames shown at once, smoothed with a 100-frame window.

To reveal the structural stability of different structures of RGD-containing liganded integrins, we first analyzed the root mean square deviations of different domains of integrin α_v_β_3_ systems during equilibration (Figure 2). RMSD represents the root mean square deviation of Cα atoms. The time–RMSD profiles of the global structure were smooth and then leveled off at ~2 Å, indicating that equilibrium had been reached (Figure 2A). The time–RMSD profiles of the global were smoother and remained at a similar level in both cyclic and linear RGD systems, whereas small fluctuations of the β-propeller and βA domain were observed in the linear system (Figure 2B,C). In particular, the trends of RMSD at the RGD-containing segment were obviously different (Figure 2D), suggesting that the structural difference is commonly located in the RGD region. Large-amplitude fluctuations may play a vital role in inhibiting the ligand from stable binding to the active-site site. To make clear the reason for the large-amplitude fluctuations, time series of the Cβ atom distance between Arg^RGD^ and Val^RGD^ were calculated (Figure 2E). Obviously, the fluctuations of the distance in the linear system were large, ranging within 6~11 Å, and the fluctuation trend was consistent with the trends of RMSD at the RGD-containing segment along the simulation time. Meanwhile, from the trajectory of the linear RGD ligand as time changed from dark to light grey, we observed that the distance at the ends of the linear RGD residues increased gradually and a little bit of rotation happened in the side chain of the benzene ring (Figure 2F). Thus, the linear RGD form induced local destabilization of the system due to its flexibility, whereas according to the rigidity of the ring structure, the cyclic peptides were more stable.

**Figure 3:**
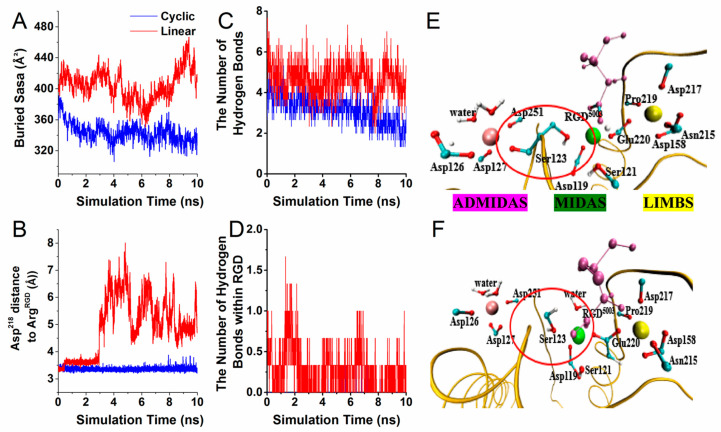
The buried solvent accessible surface area and the major interactions during equilibration. Each curve presented an average of data from three independent runs. (**A**) The buried solvent accessible surface area (SASA) during equilibration. (**B**) The distance between Asp^218^ and Arg^RGD^, (**C**) Time series of the number of hydrogen bonds for receptor–ligand interaction. (**D**) Time series of the number of hydrogen bonds for the RGD interactions. (**E**,**F**) Coordination spheres of the three important metal ions in the (**E**) cyclic RGD liganded system and (**F**) linear RGD liganded system. Atoms are shown with a CPK representation (carbon atoms are cyan, oxygen atoms are red, and hydrogen atoms are white), and a portion of integrin is shown as a yellow ribbon. The ADMIDAS (purple), MIDAS (green), and LIMBS (yellow) ions are shown in a VDW representation.

Integrin can form strong noncovalent bonds with RGD-containing peptides that bind to a shallow crevice rather than into a deep binding pocket, which is not well shielded from attacks by water molecules. To explore the influence of such differences on the binding surface, we tested the SASA value of the buried surface. It was found that the buried SASA value in the linear RGD complex was approximately 420 Å^2^, whereas the buried SASA value in the cyclic RGD system was markedly decreased (~340 Å^2^) (Figure 3A). It was speculated that due to the flexibility of the linear RGD, the steric conformation became more relaxed, and the contact area was larger, thereby blocking the access of free water molecules to the most critical RGD-α_v_β_3_ interaction surface.

Simultaneously, the major interactions between integrin and RGD peptides in the equilibrium processes were investigated. It was found that the salt bridge between Arg^RGD^ and Asp^218^ remained intact throughout the cyclic RGD simulation, whereas the occupancies were significantly decreased in the linear system (Figure 3B,G). Hydrogen bond counting showed that compared with the cyclic RGD peptide, several hydrogen bonds (Asp^218^-Arg^RGD^, Ser^123^-Asp^RGD^, Arg^216^-Asp^RGD^) had significantly higher occupancies in the linear RGD system (Table 1). Quantitatively, the number of hydrogen bonds between integrin and RGD peptides was approximately 6 and 2–4 in the linear/cyclic RGD liganded system, respectively (Figure 3C). Moreover, the linear RGD peptides could easily form intramolecular hydrogen bonds with themselves, which could not be formed in the cyclic RGD sequences (Figure 3D). Thus, it was speculated that the form of intramolecular and intermolecular hydrogen bonds may block the binding of linear RGD peptides to integrin.

The occupancies of Ser^123^-Asp^RGD^ were 0.21% and 66.81% in the cyclic RGD and linear RGD systems, respectively (Table 1). We found that one carboxylate oxygen of Ser^123^ came into contact with the MIDAS Mg^2+^ ion, and another side chain was directly coordinated with ADMIDAS in the cyclic RGD, whereas the Ser^123^ side-chain group formed two salt bridges with MIDAS ion and Asp^RGD^ in the linear RGD (Figure 3E,F). In the linear RGD, Ser^123^ bound to the Asp of RGD, except in the cyclic RGD, indicating that the cyclic RGD was far away from Ser^123^, thus presenting a smaller binding surface.

**Figure 4:**
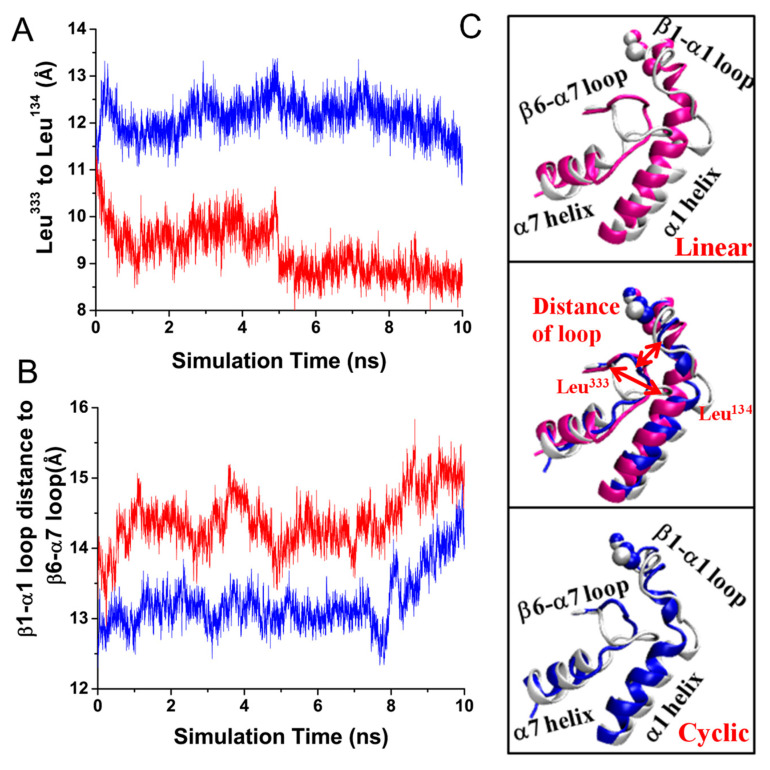
(**A**) Time series of the C_β_-atom distance between Leu^134^ of the α1 helix and Leu^333^ on the β6 strand. (**B**) Time series of the distance between the centers of mass of the β1-α1 loop and the β6-α7 loop. (**C**, top and bottom) The superposition of the equilibrated complex and the unliganded structure (silver). Each conformation was an average of the last 10 ps simulation times with three runs during equilibrating. (**C**, middle) Both of the equilibrated complexes were closely aligned with the conformation of the unliganded structure (silver). The selected regions are shown in a NewCartoon representation. Red arrows identify the region where the β1-α1 loop met the β6-α7 loop, and the region where Leu^134^ of the α1 helix contacted with Leu^333^ of the β6 strand and α7 helix during T-junction formation.

We further analyzed the dynamic trajectories during equilibration to estimate whether the different conformational features of the ligand caused a local structural change in integrin α_v_β_3_. Vogel et al. reported that ligand binding could induce the activating integrin α_v_β_3_ conformational change via the formation of the T-junction between the middle of the α1 helix and the top of the α7 helix [13]. Similarly, we measured the distance between Leu^134^ of the α1 helix and Leu^333^ on the β6 strand to identify the formation of T-junctions. This distance is shown in Figure 4A. The comparison between the linear and cyclic RGD revealed that there was a considerable decrease in distance in the linear RGD, whereas the cyclic RGD remained nearly identical throughout the simulation time. Corresponding to a decrease in the C_β_-atom distance, the bidirectional allosteric signal process was accompanied by an increase in distance between the β1 and α1 and β6–α7 loops. A similar increase in the separation between the β1–α1 and β6–α7 loops was observed in both systems. However, the increase in distance in the linear region was obvious (Figure 4B).

These findings were further illustrated (Figure 4C) by the alignment of both the linear and cyclic RGD and the corresponding domains from the unliganded α_v_β_3_ integrin after 10 ns of equilibration. Restoration of the β1–α1 loop was found in both the linear and cyclic liganded structures. In addition, another characteristic helical structural alteration was the inward movement of the α1 helix. Moreover, the modifications were more apparent in the linear RGD-occupied integrin α_v_β_3_ due to the firm bond of Ser^123^-Asp^RGD^ (occupancy 66.81% vs. 0.21%) than in the cyclic RGD. The inward shift of the α1 helix has been reported to promote βA/hybrid domain hinge opening along the allosteric pathway [12,29,30]. Whether it is easier for the linear RGD-containing peptides to trigger the allosteric pathway of integrin activation requires further verification combined with other domains, such as the hybrid domain and transmembrane domains.

### 3.2. Interaction Energy of RGD Peptide Binding to Integrin α_v_β_3_

**Figure 5:**
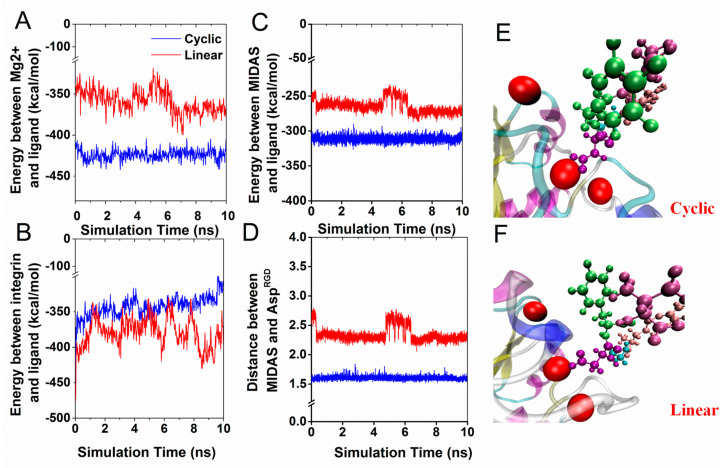
The interaction energies of RGD peptides targeted with integrin α_v_β_3_. Each curve presented an average of data from three independent runs. (**A**) Interaction energies of three Mg^2+^ ions contacting RGD peptides. (**B**) Interaction energies of the RGD peptide domain and integrin α_v_β_3_. (**C**) Interaction energies of MIDAS contacting RGD peptides. The term of interaction energy included electrostatic and van der Waals. (**D**) Time series of the distance between the mass center of two oxygen atoms of Arg^RGD^ and MIDAS ions. (**E**) The binding sites of the cyclic RGD pentapeptide and the MIDAS ion. (**F**) Binding site of the linear RGD pentapeptide and the MIDAS ion.

From the crystal structure of the cyclic pentapeptide in integrin α_v_β_3_, we observed that Asp^RGD^ coordinated with a metal ion located at the MIDAS; Arg^RGD^ formed two salt bridges with Asp^218^ and Asp^150^ in the α_v_ subunit. This phenomenon indicates that cooperative interaction between RGD and integrin α_v_β_3_ occurs not only through the neighboring polar or charged amino acids, but also through the Mg^2+^ metal ion. To elucidate the effect of different integrin domains interacting with RGD, we analyzed the electrostatic energies and van der Waals interactions between RGD and integrin based on a series of molecular dynamics simulations, and the interaction energies and the key interactions are shown in Figure 5. Our results show that the kinetic energy barrier between the metal ion Mg^2+^ and cyclic RGD was approximately −420 kcal/mol, which is higher than that observed for the linear RGD system (−350 kcal/mol) (Figure 5A). Among the three Mg^2+^ ions at MIDAS, ADMIDAS, and LIMBS, compared to the linear RGD, the interaction energies of the cyclic RGD residues interacting with the Mg^2+^ at MIDAS appeared to be much higher (Figure 5C). The fluctuation of interaction energies was consistent with the distance of the mass center of two oxygen atoms of Arg^RGD^ and MIDAS ions (Figure 5D). The most significant change was that only one of the two carboxylic oxygen atoms from Asp^RGD^ formed contact with the MIDAS ion during the equilibration of the linear system (Figure 5F), whereas both carboxylic oxygen atoms of Asp^RGD^ remained in contact with the MIDAS ion in the cyclic RGDfV (Figure 5E). Therefore, even though the total interaction energy of RGD-α_v_β_3_ was at a similar level in both systems, the larger energy fluctuation observed in linear RGD may have been the cause of structural instability in the linear system (Figure 5B).

### 3.3. Force Induced Unbinding of Liganded Integrin α_v_β_3_

**Figure 6:**
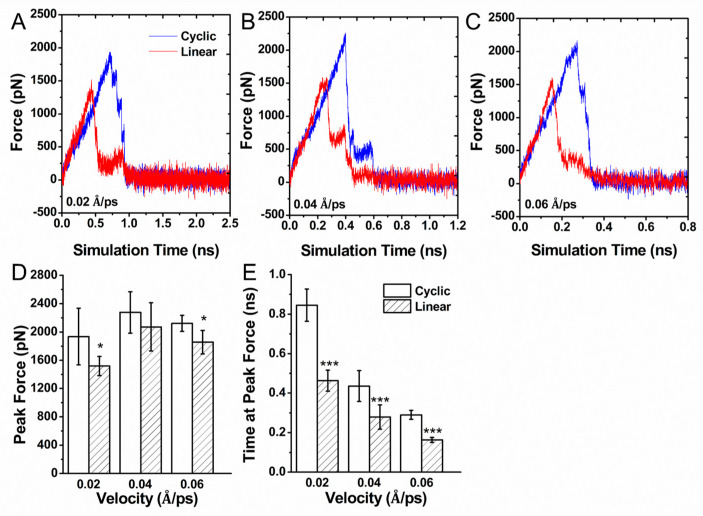
Force-induced dissociation of linear vs. cyclic RGD from integrin α_v_β_3_ by steered molecular dynamics. The force profiles were obtained from five independent simulations of pulling the ligand away from integrin α_v_β_3_ by pulling speed *v* = 0.02 Å⋅ps^−1^ (**A**), *v* = 0.04 Å⋅ps^−1^ (**B**), and *v* = 0.06 Å⋅ps^−1^ (**C**). Average peak force (**D**) and average time required to reach peak force (**E**) are indicated. *Error bars*: standard deviation from the mean; *asterisks*: statistical significance following the *t*-test (* *p* < 0.05; *** *p* < 0.01).

To map the unbinding dynamics of different RGD structures on the interaction mechanism with integrin α_v_β_3_ at the atomic level, external forces were applied to the Cα atom of the RGD^5005^ of the ligand to facilitate its binding with integrin α_v_β_3_. The pulling speed *v* = 0.02 Å⋅ps^−1^ was set based on others’ publication [31]. The rupture force profiles in the dissociative trajectories are shown in Figure 6A. At the same time, we also set up two other speeds, *v* = 0.04 Å⋅ps^−1^ (Figure 6B) and *v* = 0.06 Å⋅ps^−1^ (Figure 6C). The rupture force of pulling cyclic RGDfV out of the binding site was much larger than that of the linear RGD pentapeptide and took more time to dissociate (Figure 6D,E). The difference in the rupture force profiles implied that it was easier for the linear RGDfV ligand to unbind to the active site and much quicker to dissociate than the cyclic RGDfV ligand. The results show good agreement with the experimental evidence that linear RGD-containing peptides have a short circulation half-life and are more susceptible to chemical degradation [20,32,33,34].

### 3.4. Characterization of Binding Pathway

After the unbinding simulation of the integrin α_v_β_3_-RGD complex, we simulated the binding process between integrin α_v_β_3_ with linear RGDfV and cyclic RGDfV. Steered MD simulations were performed to enforce the binding of the RGD-containing ligand to the binding site, as they were unable to effectively and suitably ligate in free MD simulations. For this purpose, the pushing direction by loading force was chosen along the vector pointing from the side-chain oxygen atom OD2 of Asp^RGD^ to the MIDAS ion, the key RGD-integrin-binding contact. The force-induced binding test was applied at a constant pulling velocity of 0.1 Å⋅ps^−1^, and the spring constant was set to 2 kcal/(mol·Å^2^). Ten separate SMD simulations were conducted for each system. However, only two of these trajectories entered the active site within a short simulation time, whereas eight of these simulations successfully recovered the bound pose in the binding site in the cyclic RGDfV.

**Figure 7:**
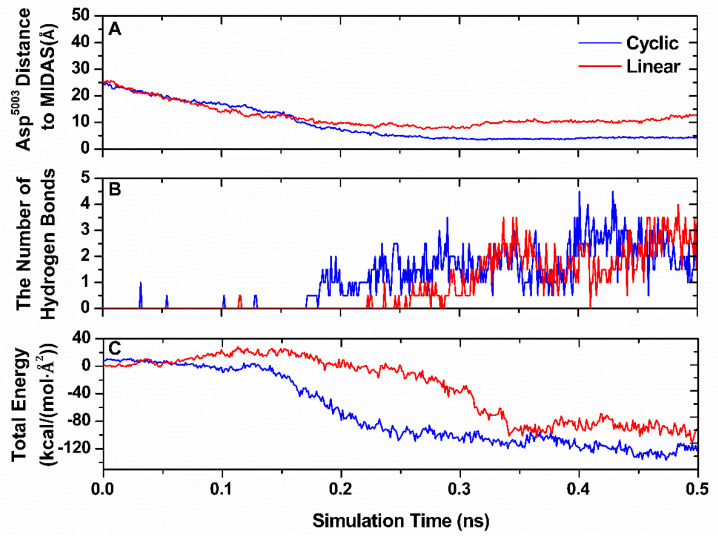
Binding pathway simulation. The distance from the oxygen atom OD2 of Asp^RGD^ to the MIDAS ion (**A**), numbers of hydrogen bonds (**B**), and interaction energies between the RGD peptide and integrin α_v_β_3_ (**C**) vs. time in the process of pushing RGD-containing peptides into the active binding site. Each curve presents an average of data from two independent runs. The term “interaction energies” refers to the total number of electrostatic and VDW interactions.

The interaction features were examined to assess the behavior of the binding process (Figure 7). Compared with the cyclic RGDfV, the distance between the side-chain oxygen atom OD2 of Asp^RGD^ and the headpiece of integrin α_v_β_3_, which was the most vital bond in the complex, was larger in the linear system (Figure 7A), implying that the interaction was much weaker for the linear system. The formation of hydrogen bonds occurred continuously while moving along the binding site (Figure 7B). The final number of hydrogen bonds was greater than that of cyclic RGD. The more hydrogen bonds, the stronger the binding forces. The linear RGD peptide may have arrived at its binding site slowly. The interaction energy profile during the binding process is shown in Figure 7C. The energies increased along both RGD peptides, pushing into the active binding site. The lowest valley was produced in the cyclic RGD system. The corresponding time was also much shorter. The results indicate that it was easier for the cyclic RGD-containing peptide to bind to the active site than the linear RGD peptides.

## 4. Discussion

This study attempted to explore the effect of the RGD structure (cyclic or linear) on integrin α_v_β_3_. Our simulations provided insight into the mechanism to explain why cyclic RGD improves tumor-targeting efficacy at the atomic level. Since the amino acids flanking the RGD sequence could alter the mechanical stability and other properties, cyclic RGDfV and linear RGDfV pentapeptides with the same residues interacting with integrin α_v_β_3_ were set up for comparison. Our data indicate that the linear RGD peptides have a higher flexibility (Figure 2D), and larger amplitude fluctuations may play a negative role in the binding or unbinding process of the α_v_β_3_-RGD interaction. Because of the flexible structure of the linear RGD, except for the linear RGD that can form a combination that was reported in [32], it was easier to form hydrogen bonds with integrin α_v_β_3_ than with cyclic RGD (Figure 3C). However, the linear RGD could interact with many residues at the ligand-binding site. Therefore, its ability to accurately locate the critical site was significantly reduced. Cyclic RGD had strong localization ability, and both the binding energy and binding efficiency with Mg^2+^ were outstanding (Figure 5A and Figure 7A). Thus, when considering the synthesis of RGD-targeting drugs, cyclic RGD peptides should be considered for their stability and effectiveness, and it is true that many drugs have been designed in recent years based on cyclic RGD, including Cilengitide [35,36].

Most interestingly, we found that the Ser^123^-Asp^RGD^ interaction could improve the formation of α1 the α7 T-junction. The formation of T-junctions may promote the βA/hybrid domain hinge opening in the integrin activation process. The residue Ser^123^, located at the top of α1 helix and close to the ligand-binding site, was conducive to the inward and upward movement of the α1 helix interacting with Asp^RGD^. The Ser^123^-Asp^RGD^ bond may play a critical role in the allosteric pathway. The findings of our study reveal that in linear RGD, the occupancy of the Ser^123^-Asp^RGD^ interaction is larger than that of cyclic RGD (Table 1). Considering the high sensitivity to minor structural perturbations along the allosteric pathway, allosteric activation requires further verification.

Previously, Yu et al. reported that the targeted recognition between RGD peptide and integrin α_v_β_3_ was mainly driven by electrostatic interactions [32]. Indeed, we were able to confirm that the interaction energy of the complex mainly involved electrostatic interactions between the residues in RGD and the MIDAS ion in integrin α_v_β_3_. However, van der Waals forces are also non-bonding forces; therefore, they are included in the energy values. Yu et al. mentioned that the interaction between RGD and ions at the ion-binding site is the mechanism of the interaction between RGD and the ion-binding site, which is consistent with the interaction between RGD and Mg^2+^ mentioned in our study. We also showed that the interaction between ions and RGD was stronger in cyclic RGD than that in linear RGD. The evidence comes from the energy level (Figure 5A) and the accuracy and velocity of interaction (Figure 7A,B). The explanation of the distinct differences is whether the carboxylic oxygen of Asp^RGD^ remains in contact with the MIDAS ion. It was shown that the oxygen atoms of Asp^RGD^ contributed significantly to the electrostatic effect on the MIDAS cationic ligands. Furthermore, the cyclic RGD resulted in a much greater decrease in the buried SASA than in the linear RGD ligand (Figure 3A). It was possible to stabilize the interaction by shielding the critical Asp^RGD^-MIDAS interaction from attacks by free water molecules in the cyclic RGD. This would enhance the mechanical stability of protein–protein interaction [17]. Our simulations provide a valid explanation for the experimental result that cyclic peptides are more stable at the atomic level.

In this study, the binding and unbinding properties of cyclic RGDfV and linear RGDfV interacting with integrin α_v_β_3_ were obtained. The rupture force of the cyclic RGD RGDfV-α_v_β_3_ complex to dissociate was much larger than that of the linear RGD pentapeptide, and the corresponding time at the peak force was also greater than the time required to pull the linear RGDfV out of the active binding site (Figure 6). The difference in forced dissociation tests suggests that the barrier along the unbinding pathway in the cyclic RGD was much larger than that in the linear RGD system. The results indicate that the binding affinity between cyclic RGDfV and integrin α_v_β_3_ was higher than that of linear RGDfV peptides. This is consistent with the experimental evidence that cyclic RGD is commonly employed to improve the binding properties of RGD peptides because of the rigidity of the ring structure [10,37]. For the RGD pentapeptide entry process, the linear RGD-containing peptides easily formed hydrogen bonds because of their flexibility, and hydrogen bonds negatively impacted the ability of linear peptides to bind integrin α_v_β_3_ accurately. Thus, they took much more time to bind to the active site than cyclic RGD (Figure 7). Additionally, the interaction energy in binding integrin α_v_β_3_ exhibited greater potential energy in cyclic peptides in a short simulation time. This distinct difference implies that it is easier for cyclic peptides to bind to the active site than to linear peptides. This could be the reason that most cyclic RGD-containing peptides have a longer circulation survival life, which results in ideal effects on treatment [38].

Recently, researchers have not only found that cyclic RGDs have important pharmaceutical value, but that polycyclic RGDs are also gaining increasing attention. For example, tetrameric cyclo (DKP-RGD) ligands have the potential to improve tumor targeting for diagnosis and therapy [39]. Therefore, there is still much room for research on the medicinal value of RGD.

## 5. Conclusions

Owing to the application value of integrin–RGD interaction in the design of oncology drugs, an increasing number of researchers are focusing on the mechanism of interaction between integrins and RGD. In our study, we found that the linear RGD peptides triggered the instability of the configurational changes due to their flexibility, and the linear RGD peptides formed their own combination more easily than cyclic RGD. The main interaction energy between Mg^2+^ and cyclic RGD was much stronger than that of the linear RGD system due to the well shield lessening attacks by free water molecules. In addition, our results show that it was easier for linear RGDfV to leave the active site and more quickly than the cyclic RGDfV ligand, whereas it was more difficult to enter the appropriate active binding site in linear RGD. The Ser^123^-Asp^RGD^ bond may play a critical role in the allosteric pathway. In conclusion, our study may provide new ideas for new drug creation and development in the treatment of tumors.

## Figures and Tables

**Table 1:** The occupancies of interactions at the RGD peptide/integrin interfaces of both simulation systems.

	Residues	Cyclic	Linear	Residues	Cyclic	Linear
Hydrogen Bond	α_v_-TYR^178^&Arg^5001^	6.05	0.07	β_3_-TYR^122^&ASP^5003^	6.75	2.40
	α_v_-ALA^213^&Arg^5001^	7.40	3.87	β_3_-SER^123^&ASP^5003^	0.21	66.81
	α_v_-GLN^214^&Arg^5001^	0.20	17.57	β_3_-ASN^215^&ASP^5003^	11.20	1.99
	α_v_-ASP^218^&Arg^5001^	97.86	83.25	β_3_-ARG^216^&ASP^5003^	19.55	42.13
	α_v_-ASP^218^&GLY^5002^	0.00	16.73	β_3_-LYS^125^&VAL^5005^	0.00	8.95
	α_v_-LYS^253^&GLY^5002^	0.00	9.00	β_3_-ARG^214^&VAL^5005^	0.00	10.56
Salt Bridge	α_v_-ASP^218^&ARG^5001^	93.10	49.07	α_v_-ASP^150^&ARG^5001^	0.20	0.00

## Data Availability

No new data were created or analyzed in this study.
